# Successful endoscopic treatment of stapled J-pouch ileoanal canal anastomotic hemorrhage by argon plasma coagulation: a case report

**DOI:** 10.1186/s13256-016-1103-4

**Published:** 2016-11-03

**Authors:** Takeshi Nishikawa, Keisuke Hata, Shuntaro Yoshida, Koji Murono, Koji Yasuda, Kensuke Otani, Toshiaki Tanaka, Tomomichi Kiyomatsu, Kazushige Kawai, Hiroaki Nozawa, Soichiro Ishihara, Kazuhiko Koike, Toshiaki Watanabe

**Affiliations:** 1 Department of Surgical Oncology, the University of Tokyo, 7-3-1 Hongo, Bunkyo-ku, Tokyo, 113-8655 Japan; 2 Department of Gastroenterology, the University of Tokyo, 7-3-1 Hongo, Bunkyo-ku, Tokyo, 113-8655 Japan

**Keywords:** Ulcerative colitis, Stapled anastomosis, Argon plasma coagulation, Case report

## Abstract

**Background:**

Continuous lower gastrointestinal hemorrhage is a rare condition, but it often requires proper management. We report a case of a patient with gastrointestinal hemorrhage 18 years after stapled J-pouch ileoanal canal anastomosis who was successfully treated with argon plasma coagulation.

**Case presentation:**

Our patient was a 54-year-old Japanese man who had developed ulcerative colitis 28 years ago. A J-shaped ileal pouch-anal anastomosis with a double-staple technique was indicated 18 years ago when the patient became refractory to the conventional medication. When he presented to our hospital, 18 years after the operation, the patient complained of faintness and fresh blood in the stool of 2 days’ duration, and was admitted for investigation. Lower endoscopy revealed that the hemorrhage was from a neovascularization area close to the site of ileal pouch-anal anastomosis. Cap-assisted argon plasma coagulation was carried out for hemostasis, and complete hemostasis was achieved without complications.

**Conclusions:**

We present a case of a patient with hemorrhage following a J-shaped ileal pouch-anal anastomosis with a double-staple technique performed 18 years ago. Argon plasma coagulation treatment was successful, suggesting the potential safety and effectiveness of colonoscopic electrocoagulation for controlling unremitting hemorrhage from a neovascularization area around a stapled ileoanal canal anastomotic site.

## Background

The use of staplers in gastrointestinal surgery is now widespread [1]. Staplers are not only time-saving but also associated with low rates of postoperative complications, with reported incidences ranging between 0 and 2.5 % [1]. Hemorrhage is a relatively rare complication, noted in 1 % of colorectal anastomoses [2, 3]. The authors of a previous report stated that pouch bleeding developed in 1.5 % of patients undergoing ileal pouch-anal anastomosis [4]. Most of bleeding occurred within 7 days postoperatively [4]. Bleeding occurring a long time after the surgery was rare.

Lower gastrointestinal tract bleeding is usually spontaneously controlled, and continuous hemorrhage is a rare event. However, when it occurs, it often requires proper management that includes accurate identification of the bleeding location and implementation of appropriate therapeutic measures. The therapeutic approach includes clinical observation with or without blood transfusion, rectal packing, angiography-assisted vasopressin infusion or embolization, colonoscopy, and eventually emergency surgery. On the one hand, angiographic techniques are effective but associated with potential risks such as bowel or myocardial ischemia and infarction. On the other hand, emergency surgery is an invasive procedure and requires anastomotic refashioning and sometimes protective colostomy. Recently, endoscopic treatment has been proven effective in achieving hemostasis of bleeding from the stapled colorectal anastomosis [1, 2, 5–7]. We report a case of a patient with hemorrhage following J-shaped ileal pouch-anal anastomosis with a double-staple technique performed 18 years ago who was successfully treated with argon plasma coagulation (APC).

## Case presentation

A 54-year-old Japanese man who had been diagnosed as ulcerative colitis 28 years ago and had been treated with conventional medications, including corticosteroids, presented to our hospital. Eighteen years ago, his colitis had become refractory to conventional medication, and J-shaped ileal pouch-anal anastomosis with a double-staple technique was performed. In the postoperative period, diarrhea and blood in the stool were observed, and a diagnosis of pouchitis was made. The patient was treated with metronidazole. Thereafter, he had repeated recurrence episodes of pouchitis. Endoscopic surveillance was regularly performed to rule out dysplasia or cancer. Upon presentation to our hospital, 18 years after the operation, he was admitted with complaints of faintness and fresh blood in the stool of 2 days’ duration.

**Fig. 1:**
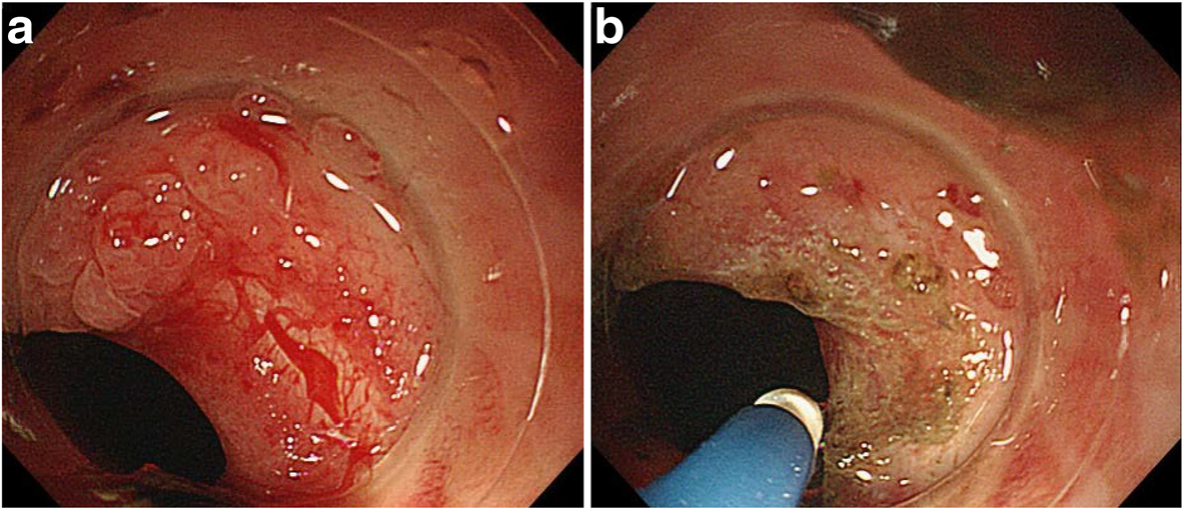
**a** Colonoscopy revealed bleeding from a neovascularization area close to the site of the patient’s J-shaped ileal pouch-anal anastomosis. **b** Two sessions of brief pulse argon plasma coagulation at a power setting of 50 W and an argon flow rate of 1.4 L/minute were performed to control the unremitting hemorrhage from the neovascularization at the anastomotic site

The patient’s vital signs were stable after an intravenous saline drip, with blood pressure of 90/56 mmHg, heart rate of 86 beats/minute, and body temperature of 36.3 °C. His laboratory data revealed a decreased hemoglobin level of 11.2 g/dl, a normal white blood cell count of 5.8 × 10^3^/μl, a normal C-reactive protein level at 0.04 mg/dl, and a normal erythrocyte sedimentation rate at 6 mm/h. A digital rectal examination revealed a large amount of blood in the pouch. Lower endoscopy was performed to confirm the origin of the hemorrhage, which revealed the hemorrhage to be from an area of neovascularization close to the site of the patient’s ileal pouch-anal anastomosis (Fig. 1a) but no evidence of pouchitis, although a biopsy was not taken, to avoid promoting the bleeding. Cap-assisted APC was carried out with a clear cap over the tip of the colonoscope for hemostasis. Two sessions of brief pulse APC at a power setting of 50 W and an argon flow rate of 1.4 L/minute were performed (Fig. 1b). The APC probe was moved close to the lesion site, and cauterization was performed carefully to avoid contact with the stapled ileal pouch-anal anastomosis. APC cauterization was performed several times to enable stoppage of bleeding, and complete hemostasis was achieved without causing pain or complications. Three months later, the patient was admitted to our hospital with a small amount of fresh blood in the stool again. Although lower endoscopy revealed no active hemorrhage, electrocoagulation was carried out in the most suspicious area, where there was an area of neovascularization close to the site of previous cauterization. After that, the patient returned for a follow-up visit, and he reported being without bleeding for 8 months.

## Discussion

Endoscopic therapies such as submucosal drug injection, appliances such as clips, and electrocoagulation such as APC [1, 5] for hemostasis of bleeding from stapled anastomosis have been reported. These treatments have obvious advantages in terms of less invasiveness for the patients, avoidance of surgery and the associated complications, no requirement for general anesthesia, and direct visualization of the bleeding site, and they are likely to be cost-saving in terms of both procedural costs and overall length of hospital stay.

APC has been available in flexible endoscopy since 1991, and it has been applied in the treatment of various conditions, such as hemorrhage, malignant and benign tumors, tissue ingrowth and overgrowth of stents, and angiodysplasia [8]. The advantage of APC is the controllable depth of coagulation (0.5–3 mm) [8]. Another known benefit of APC is a device used for noncontact thermal coagulation of tissue. The probe can be applied axially and radially, allowing tangential coagulation of lesions around rectal bends. A further advantage in treating active hemorrhagic lesions is that blood may be blown off the tissue surface by the argon flow, resulting in a direct effect of the electrocoagulation current on the bleeding lesion. The argon jet keeps oxygen away from the target area and prevents tissue carbonization and smoke production [9]. However, minor complications of APC include gas bloating, as well as transient abdominal or anal pain in 10 % of patients [10]. In addition, major complications include bowel explosion with perforation, rectovaginal fistulae, chronic rectal ulceration, and stricture. These major complications of APC are rare and are reported to be less than 0.3 % [10]. Although APC is reported to be associated with limited complications, major ulcers, strictures, and fistulas have been reported, and careful monitoring and attention are necessary with APC.

In our patient, cap-assisted APC was carried out with a clear cap over the tip of the colonoscope, as previously reported [11, 12]. The use of a transparent cap at the tip of the colonoscope allows direct viewing of the anal canal without obstructing the lumen. Stability is maintained by placing the tip of the colonoscope against the anal canal, thus allowing safe APC. Because of the presence of the cap, the colonoscope remains close to the lesion, but not too close to it.

Some previous reports demonstrated successful colonoscopic electrocoagulation to control hemorrhage from a stapled colorectal anastomosis [2, 5]. While no study has been conducted to evaluate the effect of electrocoagulation on the staple line of the anastomosis, Cirocco and Golub reported that an anastomotic fistula developed in one of six cases treated with colonoscopic electrocoagulation [2], and Law *et al.* reported a case of perforation at the metallic stapled line, the location of the blind end of the colonic L-pouch remote from the area of APC treatment [10]. Owing to the small number of cases reported in the literature, it is difficult to assess whether this procedure potentiates this complication. In such a situation, Cirocco and Golub also reported a case of an anastomotic fistula in a patient with postoperative hemorrhage from a stapled colorectal anastomosis who had been treated with blood transfusion only [2]. So, further studies are needed to evaluate if colonoscopic electrocoagulation is a safe option to control hemorrhage from a stapled anastomosis. As Fujishiro *et al.* suggested in their report, the most important parameter affecting tissue damage is pulse duration under the generally applied condition [8]. The changes of power and argon plasma flow caused only minor differences in tissue damage [8]; thus, the duration of the pulse must be taken into account when performing APC. Although Manner *et al.* reported that APC with prior submucosal fluid injection reduced coagulation depth [13], in our patient it was difficult to perform APC with prior submucosal fluid injection because of the anastomotic site hemorrhage.

## Conclusions

We present a case of a patient with hemorrhage following a J-shaped ileal pouch-anal anastomosis with a double-staple technique performed 18 years ago, which was successfully treated by APC, suggesting the potential safety and effectiveness of colonoscopic electrocoagulation in controlling unremitting hemorrhage from a neovascularization area around the stapled ileoanal canal anastomotic site. Because fistula formation and bowel perforation have been reported after this procedure, appropriate selection of patients and prudent use of APC are requisites for achieving safe hemostasis.

## Abbreviation

APC: Argon plasma coagulation
